# A Case for Public-Private Partnerships in Health: Lessons From an Honest Broker

**Published:** 2009-03-15

**Authors:** Robert McKinnon

**Affiliations:** Yellow Brick Road

## Introduction

Collaboration between the private and public sectors has produced mixed results. Some initiatives seem better suited to this model. For example, partnerships in national defense and space exploration have produced benefits to both sectors, although not without some controversy. Conversely, with issues such as education and health, the road to success has been much rockier. As an honest broker who has worked effectively to bridge the public and private worlds, I have come to understand the barriers to successful collaboration through my work with Fortune 500 companies, large foundations, small nonprofit organizations, and state and federal governments. In this essay, I explore these barriers and cite examples showing when they were successfully bridged through work with organizations such as the Centers for Disease Control and Prevention (CDC), the Food and Drug Administration (FDA), and the Robert Wood Johnson Foundation.

## Perspectives

In Robert Putnam's *Bowling Alone* (1), the author describes several purposes for partnerships in general that translate well to the potential dynamic for public-private partnerships. The first, "Engage with another organization or entities in order to achieve goals that neither partner could accomplish alone," is probably the most relevant for this discussion. Many health issues, if not addressed, will have serious implications for both the public and private sectors for generations to come. The health of the citizenry and the health of the economy are, in many ways, inseparable. Considering the growing severity of issues such as childhood obesity and rising health care costs, neither the public nor the private sector can address the issues alone but must do so jointly. Given the simplicity of this logic, why have there not been more examples of successful collaboration, and how can players in the public and private sectors address the barriers that stand in the way of more creative partnerships?

Chief among the barriers that prohibit successful collaborations is a lack of appreciation for the different roles that the public and private sectors play in society. Many in the public health arena distrust business, which they see as having a blinding commitment to maximizing profit. They do not see that corporations are legally bound to their shareholders to solely pursue this end. Conversely, the private sector often sees public health as obstructionist, with rules and regulations that tie the hand of the free market and in doing so, decrease global competitiveness. They do not see the obligation that government has to protect its citizens as outlined in the Constitution. These are obviously broad generalizations, but they are sadly reflected in my experience. These simplistic perspectives translate into a general unwillingness to engage in partnerships in the first place, and when partnerships are initiated, the process is marred by skepticism of the other's motives at each step. I have been in far too many meetings with representatives from these different worlds where a mutual lack of respect was palpable and blocked any potential progress.

A more realistic interpretation of the situation would begin with a simple acceptance that the public and private sectors have different but equally valid reasons for desiring the same outcome, a healthier citizenry. The problem is that these 2 sectors behave very differently, each possessing distinct drivers, different frames for how they view the world, different cultures in which they operate, and different languages when referring to the world of partnerships. For example, consider worker health. Public health officials talk of outcomes and interventions, package their materials by disease state (eg, heart disease, diabetes), and provide companies with voluminous information that too often is not presented in a way that can lead to action. Employers need solutions to insurance premium increases that seem unending, workers' compensation costs that continue to rise, and a workforce that is becoming more and more unhealthy. Both parties would benefit from a healthier workforce, but both begin on different pages. Although these barriers are substantial, they can be easily addressed to create partnerships that are mutually beneficial.

## Implications for Practice

Below are 6 key tenets that have proved instrumental to developing mutually beneficial public-private partnerships.

- *Accept that "doing good" and "making money" are not mutually exclusive ideas but rather potentially complementary ends*. One example of a positive partnership addressing the differences between the public health goal of disease prevention and the corporation goal of increasing profitability is an initiative undertaken by CDC concerning workplace health. Too often, materials on disease states or risk factors relevant to workplace health are in formats, lengths, and language based on what a health practitioner would want to know and not what an employer needs to know. The information emphasizes the health benefit to the employee, and only tangentially is the economic effect covered. CDC's goal is to more clearly and succinctly communicate the positive economic benefits of a healthier employee, and is organized by economic benefits, such as improved worker productivity and lower health insurance premiums. The information is packaged in a format that more closely resembles the *Wall Street Journal* than the *New England Journal of Medicine*, with the company's chief executive or financial officer in mind instead of the human resources director. By appreciating each other's motives and perspectives, partners can create partnerships in which it is clear that both parties can meet their objectives.
- *Leverage differences.* When the public and the private sectors appreciate each other's distinct roles, they are in a better position to leverage and negotiate toward those differences. A case in point is a recent initiative undertaken by the FDA to improve nutritional literacy among children. Launched in 2007 and equipped with modest financial resources, the FDA's initiative sought to empower preteen children to use the food facts labels on packaging to make healthier food choices. Partnering with private-sector agencies to create a youth-directed brand, Spot the Block, the FDA then leveraged the market by creating an open bidding process, which would result in 1 media company having an exclusive relationship with the FDA on this project. Although the financial element was attractive, it was by understanding the nature of the competitive media environment and the importance of exclusivity that the FDA was able to get maximum value from its media partner for the creation of materials and airtime to promote the Spot the Block brand.
- *Do not let the perfect be the enemy of progress.* Fear of failure or criticism has ended many potential partnerships before they could begin. In the example above, both parties considered the potential implications of such a partnership. From the FDA perspective, partnering with a media company could cause concern among advocates that they were "sleeping with the enemy," given that these same children-focused media outlets overtly promoted unhealthy foods and implicitly endorsed sedentary behavior. The media company was concerned that its primary source of revenue, food advertisers, would be alienated by a campaign that called on kids to pay attention to the nutritional quality (or lack thereof) of their products. Rather than allowing these concerns to turn contentious, both parties were empathetic to each other, and they created the parameters of the partnerships with the other's concerns in mind. One specific solution was to ensure that no advertisement of a food of low nutritional quality would appear near any of the Spot the Block messages, thus satisfying the concerns of both the FDA and the network's advertisers. The net result was an award-winning campaign whose results have exceeded expectations and a positive working relationship.
- *Design well*. In their book *Nudge: Improving Decisions about Health, Wealth, and Happiness*, Richard Thaler and Cass Sunstein wisely posit that 1) all of our choices matter and therefore 2) we are all architects of choice and should always design our choices with the desired outcome in mind (2). They point out that default or "no choice" is ironically a choice. Therefore, when constructing the parameters of a partnership, great care must be taken in designing the partnership with the best possible outcome in mind. This starts with the label assigned to the collaboration. Words and language are precious because each person ascribes different frames of reference to them. One person's partnership is another's alliance. It must be clear up front what each party expects to get out of a collaboration, by asking each other the difficult questions and answering them honestly: What do you have?, What do you need?, Why do you need it?, What will you get out of this?, Why partner with us?, and How long do you want to be together?
- *Manage expectations.* Every relationship has its difficulties. And, assuredly, so too will every public-private partnership. The work is too complex, the issues too important, the outcomes too critical to each party to avoid problems. Perhaps no issues and no public-private partnerships have been more difficult to navigate in the last 10 years than those involving childhood obesity. In the first efforts to address childhood obesity, about 1998, there was the expected finger-pointing and the responsibility shell game whose only guaranteed result was inertia. As someone who was involved in these efforts, having testified to both the Federal Trade Commission and the Institute of Medicine, I can attest to the lack of progress in the early years. Each of these meetings was attended by the same groups of well-intentioned people speaking from their own constituents' point of view, often placing blame for the lack of progress on another party and failing to appreciate the others' points of view. However, as the severity of the issue has become more obvious and the need for action more palpable, slowly there has been movement. Although some at the center of these discussions on childhood obesity may say that it has come too slowly and perhaps a little too late, still progress has been made. Increasingly, food manufacturers, advocates, media companies, and government officials are creating better practices and better products; the elimination of *trans* fats, smaller portion sizes, and better labeling systems have all facilitated healthier choices for American consumers.
- *Start with a bigger shared objective.* Those in the private sector passionately labor toward their objectives while those in the public sphere do the same. Even partners can fail to see the true connection between these 2 worlds and to embrace the possibility of a shared objective. When CDC's VERB campaign was launched, the communications team followed many of the above tenets when entering into the many partnerships with the private sector. Perhaps the most important variable in assuring that these many partnerships would bear fruit was a simple and very human element incorporated early on. As part of the initial meeting process, the team gathered and talked candidly and passionately about what they were trying to do and the critical role that the private sector could play in trying to reverse the growing epidemic of obesity and the corresponding decline in physical activity among America's youth. The campaign illustrated with clarity and humility the definition of partnership, that there was a goal that was important to us all but that neither could accomplish alone. Today, VERB is widely acknowledged as one of the most successful public health campaigns of its kind. Unprecedented increases in physical activity among youth were just 1 measure of its success. Another is the legacy it leaves as having spawned so many valuable collaborations with the private sector. These collaborations have in turn extended their commitment to the cause of ending the epidemic of childhood obesity, beyond their valued contributions to the VERB campaign.

## Conclusion

Henry Ford said, "Coming together is a beginning. Keeping together is progress. Working together is a success." We have seen some progress in the partnership of the public and private sectors in the past several years, like some of the examples cited in this article. Although many good partnerships have the promise of progress, we have just touched the surface of what could be done with better collaboration. What will be the public-private partnership equivalent in health to a man landing on the moon? Ending childhood obesity? Creating a more sustainable workplace for employees and their employers? Achieving lasting health care reform? The promise remains. The potential is great.
